# Relationship between digestive tract colonization and subsequent ventilator-associated pneumonia related to ESBL-producing Enterobacteriaceae

**DOI:** 10.1371/journal.pone.0201688

**Published:** 2018-08-08

**Authors:** Marion Houard, Anahita Rouzé, Geoffrey Ledoux, Sophie Six, Emmanuelle Jaillette, Julien Poissy, Sébastien Préau, Frédéric Wallet, Julien Labreuche, Saad Nseir, Benoit Voisin

**Affiliations:** 1 CHU Lille, Critical Care Center, Lille, France; 2 Univ. Lille, Faculty of Medicine, Lille, France; 3 CHU Lille, Centre de Biologie et de Pathologie, Lille, France; 4 CHU Lille, Clinique de Santé Publique, Plateforme d'Aide Méthodologique, Lille, France; National Yang-Ming University, TAIWAN

## Abstract

**Background:**

Ventilator-associated pneumonia (VAP) is the most common ICU-acquired infection. Recently, the incidence of extended-spectrum beta-lactamase producing Enterobacteriaceae (ESBLE) has substantially increased in critically ill patients. Identifying patients at risk for VAP related to ESBLE could be helpful to improve the rate of appropriate initial antibiotic treatment, and to reduce unnecessary exposure to carbapenems. The primary objective was to identify risk factors for VAP related to ESBLE. Secondary objective was to determine the impact of ESBLE on outcome in VAP patients.

**Methods:**

This retrospective study was conducted in a single mixed intensive care unit (ICU), during a 4-year period. All patients with confirmed VAP were included. VAP was defined using clinical, radiologic and quantitative microbiological data. VAP first episodes were prospectively identified using the continuous surveillance data. Exposure to different risk factors was taken into account until the diagnosis of ESBLE VAP or until ICU discharge, in patients with ESBLE VAP and VAP related to other bacteria, respectively. In all patients, routine screening for ESBLE (rectal swab) was performed at ICU admission and once a week. Patients with ESBLE VAP were compared with those with VAP related to other bacteria using univariate analysis. All significant factors were included in the multivariate logistic regression model.

**Results:**

Among the 410 patients with VAP, 43 (10.5%) had ESBLE VAP, 76 (19%) patients had polymicrobial VAP and 189 (46%) had VAP related to multidrug resistant bacteria. Multivariate analysis identified prior ESBLE colonization of the digestive tract as the only independent risk factor for ESBLE VAP (OR [95% CI] = 23 [10–55], p < 0.001). Whilst the positive predictive value of ESBLE digestive colonization was low (43.6%), its negative predictive value was excellent (97.3%) in predicting ESBLE VAP. Duration of mechanical ventilation (median [IQR], 28 [18,42] vs 23 [15,42] d, p = 0.4), length of ICU stay (31 [19,53] vs 29 [18,46] d, p = 0.6), and mortality rates (55.8% vs 50%, p = 0.48) were similar in ESBLE VAP, compared with VAP related to other bacteria.

**Conclusion:**

Digestive tract colonization related to ESBLE is independently associated with ESBLE VAP. Its excellent negative predictive value suggests that patients without ESBLE colonization should not receive carbapenems as part of their initial empirical treatment to cover ESBLE.

## Introduction

Ventilator-associated pneumonia (VAP) is the most common nosocomial infection in the intensive care unit (ICU) [1–3]. VAP is associated with high morbidity and attributable mortality, ranging from 10 to 30% in different studies [4–7]. VAP also leads to prolonged duration of mechanical ventilation and length of stay [7–9].

As inappropriate initial antimicrobial treatment is an important risk factor for mortality, the choice of empiric antimicrobial treatment for critically ill patients is often challenging [10,11]. Infections involving multidrug-resistant (MDR) bacteria are common in ICU and a negative impact on mortality is reported in patients with severe infections related to these bacteria [12,13]. Among MDR bacteria, extended-spectrum beta-lactamase producing Enterobacteriaceae (ESBLE) became a serious threat. A recent meta-analysis of thirteen studies reported a rate of ICU-acquired ESBLE ranging 5–10% in the United States and Europe [14]. Previous use of beta-lactam/beta-lactamase or carbapenems and recent hospitalization were identified as risk factors for ESBLE colonization. In addition, digestive tract colonization was associated with significantly higher frequency of ESBLE subsequent infection and increased mortality [14].

According to the current guidelines, patients with risk factors for pneumonia related to MDR bacteria should receive broad-spectrum antimicrobial treatment [2,15]. Given the recent spread of ESBLE, this strategy leads to an increased use of carbapenems, considered as the treatment of choice in ESBLE infections [15–17]. Due to the risk of antimicrobial resistance, empirical treatment should be selected with caution. Thus, identifying patients at risk for VAP related to ESBLE could be useful to reduce the spectrum of initial antibiotic treatment.

We performed this retrospective study to identify risk factors for VAP related to ESBLE in the ICU. The secondary objective was to determine the impact of ESBLE on outcome of VAP patients.

## Patients and methods

### Study characteristics

This retrospective observational study was conducted during a 4-year period, From January 2008 through January 2011, in a 30-bed mixed ICU, located in the University Hospital of Lille, France. All patients with confirmed VAP were included. Only first episodes of VAP were included and were prospectively identified using surveillance of nosocomial infections. The local IRB approved the study (Comité de Protection des Personnes Nord Ouest IV, N° HP04). According to the French law, no informed consent was required because of the observational and retrospective design of the study.

### Definitions

VAP was defined as pneumonia diagnosed after 48 hours of intubation and mechanical ventilation. The diagnostic criteria for VAP included a new infiltrate on chest X-rays in conjunction with at least two of the following: core temperature ≥ 38,5°C or < 36°C, leukocyte count ≥ 10 x 10^9^/L or < 1,5 x 10^9^/L, and purulent tracheal aspirate or sputum. In addition, a microbiological confirmation was required for all patients (positive endotracheal aspirate culture ≥ 10^6^ colony-forming units (cfu)/mL or positive bronchoalveolar lavage culture ≥ 10^4^ cfu/mL) [1,2].

The following microorganisms were defined as MDR bacteria: ceftazidime or imipenem-resistant *Pseudomonas aeruginosa*, *Acinetobacter baumannii*, ß-lactamase-producing Gram-negative bacilli (ESBL) and methicillin-resistant *Staphylococcus aureus*.

### Data collection

The characteristics of all study patients were prospectively recorded at ICU admission: age, male gender, severity of illness based on Simplified Acute Physiology Score (SAPS) II, Logistic Organ Dysfunction (LOD) score, McCabe score, comorbidities (diabetes, chronic obstructive pulmonary disease (COPD), restrictive respiratory failure, chronic heart disease, cirrhosis, chronic renal failure requiring dialysis, or immunosuppression), risk factors for MDR bacteria (prior antibiotic exposure defined as antibiotic treatment received for at least 48h within the 30 days preceding ICU admission, hospitalization prior to ICU > 48 h, nursing-home resident) [2], infection at admission, location before ICU admission, admission category, main admission diagnosis (recent surgery, acute exacerbation of COPD, acute respiratory distress syndrome (ARDS), shock, pneumonia, congestive heart failure, neurologic failure, poisoning, cellulitis, cardiac arrest, and others). During ICU stay, the following data were prospectively collected: occurrence of VAP, duration of mechanical ventilation, length of ICU stay and ICU mortality. Data related to antibiotic exposure and ESBLE digestive tract colonization were retrospectively collected. The use of broad-spectrum antibiotics was defined by at least 24h of the following antibiotics: Piperacillin/Tazobactam, Cefepime, Ceftazidime, quinolones active against *P*. *aeruginosa*, and Carbapenems, from ICU admission to VAP diagnosis. Selective digestive decontamination was not used during the study period.

A routine screening for ESBLE digestive colonization was performed by rectal swab at ICU admission and repeated once a week. All samples were placed on a standardized selective agar medium (Mueller-Hinton). During the study period, the technique for standard antibiotic susceptibility testing in our microbiology laboratory was a disc diffusion method. Antibiotic discs containing a cephalosporin were then added by technicians. After a 24h-incubation, the susceptibility of bacteria to antibiotics was determined by reading the diameter of inhibition. Confirmation of ESBLE was obtained with a MAST phenotypic test. An aspect of champagne cork was characteristic of extended spectrum beta-lactamase. Patients were considered as ESBLE carriers when a rectal swab returned positive.

### Statistical analysis

SPSS software (SPSS, Chicago, IL, USA) was used for data analysis. Results are presented as numbers (percentage) for categorical variables. The distribution of quantitative variables was tested and was abnormal, results are presented as median (25^th^-75^th^ interquartile). In order to determine factors associated with ESBLE VAP, patients with ESBLE VAP were compared with those with VAP related to other bacteria using univariate analysis. The Pearson’s chi-square test was used to compare qualitative variables. The Mann-Whitney *U* test was used to compare quantitative variables. The statistical significance was set at *p* < 0.05.

Multivariate analysis was used to determine risk factors independently associated with ESBLE VAP. All data from univariate analysis with *p* values < 0.05 were included in the multivariate logistic regression model. Potential interactions were tested and the Hosmer-Lemeshow goodness-of-fit was calculated.

The odds ratio (OR) and 95% confidence interval (CI) were calculated for all significant variables in univariate and multivariate analysis. Exposure to potential risk factors for ESBLE VAP was taken into account until the occurrence of ESBLE VAP or until ICU discharge in patients with ESBLE VAP or VAP related to other bacteria, respectively.

Sensitivity, specificity, positive, and negative predictive values were calculated by standard statistical methods for identified independent factors.

## Results

### Patient characteristics

During the study period, 1570 patients received mechanical ventilation for more than 48 hours and 469 patients had a suspected VAP. Forty-two patients were excluded from analysis because VAP was not documented by microbiological results. Data were not available for 17 patients. Among the 410 remaining patients with confirmed VAP, 43 (10.5%) were related to ESBLE. Among the 43 patients with ESBL VAP, 13 (30.2%) had a positive blood culture related to ESBL. No outbreak occurred during the study period. Patient characteristics at ICU admission are presented in Table 1. No significant difference was found in age, SAPS II, comorbidities and risk factors for MDR bacteria.

**Table 1:** Patient characteristics at ICU admission.

Variable	ESBLE VAP n = 43	No ESBLE VAP n = 367	*p*
**Age, years**	61 [53–74]	63 [52–72]	0.92
**Male gender**	25 (58)	268 (73)	0.04
**SAPS II**	54 [37–70]	49 [38–63]	0.28
**LOD score**	6 [4–8]	6 [3–8]	0.86
**McCabe score**			0.2
**1**	16 (37)	148 (40)	
**2**	10 (23)	88 (24)	
**3**	0 (0)	26 (7)	
**Comorbidities**			
**Diabetes**	8 (18)	65 (18)	0.88
**COPD**	14 (33)	106 (29)	0.61
**Restrictive respiratory failure**	4 (9)	55 (15)	0.32
**Chronic heart disease**	6 (14)	68 (19)	0.46
**Cirrhosis**	4 (9)	13 (4)	0.07
**Chronic renal failure**	3 (7)	16 (4)	0.44
**Immunosuppression**	14 (33)	74 (20)	0.06
**Direct admission**	15 (35)	136 (37)	0.47
**Category of admission**			
**Medical**	36 (84)	249 (68)	0.03
**Surgical**	7 (16)	118 (32)	
**Main admission diagnosis**			
**Acute exacerbation of COPD**	1 (2)	69 (19)	0.007
**ARDS**	9 (21)	33 (9)	0.01
**Shock**	24 (56)	139 (38)	0.02
**Pneumonia**	10 (23)	65 (18)	0.37
**Congestive heart failure**	0 (0)	8 (2)	0.33
**Neurologic failure**	1 (2)	27 (7)	0.22
**Poisoning**	3 (7)	35 (10)	0.58
**Cellulitis**	4 (9)	26 (7)	0.6
**Cardiac arrest**	3 (7)	45 (12)	0.3
**Others**	0 (0)	14 (4)	0.19
**Risk factors for MDR bacteria**			
Prior antibiotic exposure	14 (33)	148 (40)	0.32
**Hospitalization prior to ICU > 48 h**	25 (58)	194 (53)	0.51
**Nursing-home resident**	1 (2)	10 (3)	0.88
**Infection at admission**	40 (93)	267 (73)	0.004

Results by univariate analysis. Data are presented as number (%) or median (interquartile range). Some patients had more that one diagnosis at intensive care unit admission.

ESBLE, extended-spectrum ß-lactamase-producing *Enterobacteriaceae*; VAP, ventilator-associated pneumonia; MDR, multidrug-resistant bacteria; ICU, intensive care unit; COPD, chronic obstructive pulmonary disease; ARDS, acute respiratory distress syndrome

### Microbiological results

VAP was polymicrobial in 76 patients (19%) and related to MDR bacteria in 189 patients (46%). *Pseudomonas aeruginosa* was the most common microorganism isolated in the cultures of VAP related to MDR bacteria. *Enterobacter* and *Klebsiella* were the main ESBLE (Table 2). VAP related to ESBL represented 11.9% of all VAP related to Gram-negative bacilli. VAP was confirmed by quantitative endotracheal aspirate, and BAL in 96% and 4% of patients, respectively. No significant difference was found between different types of bacteria and duration of mechanical ventilation (data not shown).

**Table 2:** Microorganisms isolated in patients with ventilator-associated pneumonia.

**ESBLE**	43 (10)
***Enterobacter cloacae***	19 (5)
***Enterobacter aerogenes***	8 (2)
***Klebsiella pneumoniae***	11 (3)
***Klebsiella oxytoca***	3 (0.7)
***Escherichia coli***	2 (0.5)
***Citrobacter freundii***	2 (0.5)
**MDR-bacteria other than ESBLE**	146 (36)
***Pseudomonas aeruginosa***	76 (19)
***Methicillin-resistant Gram-positive***	29 (7)
***Acinetobacter baumannii***	41 (10)
**No-MDR Gram-negative bacteria**	
***Pseudomonas aeruginosa***	64 (16)
***Escherichia coli***	28 (7)
***Enterobacter cloacae***	17 (4)
***Serratia marcescens***	17 (4)
***Proteus mirabilis***	13 (3)
***Enterobacter aerogenes***	11 (3)
***Klebsiella pneumoniae***	10 (2)
***Acinetobacter baumannii***	10 (2)
***Citrobacter koseri***	8 (2)
***Klebsiella oxytoca***	6 (1)
***Haemophillus influenzae***	6 (1)
***Citrobacter freundii***	5 (1)
**Others**	10 (2)
**No-MDR Gram-positive cocci**	
***Staphylococcus aureus***	33 (13)
***Streptococcus pneumoniae***	4 (1)
**Others**	3 (0.7)
**Polymicrobial**	76 (19)

Results are numbers (%) of VAP patients with different bacteria

ESBLE, extended-spectrum ß-lactamase-producing Enterobacteriaceae; VAP, ventilator-associated pneumonia; MDR, multidrug-resistant bacteria.

During ICU stay, 297 patients (72.4%) acquired MDR bacteria. Among them, digestive tract colonization with ESBLE was found in 125 patients (30.5%). 79% of ESBLE VAP occurred in patients previously identified as ESBLE carriers (Table 3). The median [IQR] time from digestive tract colonization to VAP related to ESBL was 8 [2,13] days.

**Table 3:** Colonization related to multidrug-resistant bacteria in study patients.

Variable	ESBLE VAP n = 43	No ESBLE VAP n = 367	*P*
**MDR colonization**			
**At ICU admission**	13 (30)	50 (14)	0.003
**ICU-acquired**	41 (95)	256 (70)	0.001
**ESBLE digestive colonization**			
**Before VAP occurrence**	34 (79)	44 (12)	< 0.001
**ICU-acquired**	37 (86)	88 (24)	< 0.001

Results by univariate analysis. Data are presented as number (%).

MDR, multidrug-resistant bacteria; ICU, intensive care unit; ESBLE, extended-spectrum ß-lactamase-producing Enterobacteriaceae; VAP, ventilator-associated pneumonia.

### Risk factors for ESBLE VAP

Exposure to different risk factors for ESBLE VAP during ICU stay is reported in Table 4. No significant difference was found in median duration of mechanical ventilation before VAP occurrence (median [IQR], 13 [6,22] vs 14 [8,18] d, p = 0.4), percentage of patients with prior broad-spectrum antibiotics (84% vs 68%, p = 0.3), or percentage of patients with prior use of carbapenems (26% vs 19%, p = 0.33), between patients with ESBLE VAP, and those with VAP related to other bacteria.

**Table 4:** Patient characteristics during ICU stay.

Variable	ESBLE VAPn = 43	No ESBLE VAPn = 367	*P]]*
**Duration of mechanical ventilation, days**	13 [6,22]	14 [8,18]	0.60
**Exposure to third generation cephalosporins**	14 (33)	84 (23)	0.16
**Broad-spectrum antibiotics, n (%)**	36 (84)	248 (68)	0.3
**Carbapenems, n (%)**	11 (26)	71 (19)	0.33

Results by univariate analysis. Data are presented as number (%) or median (interquartile range).

ESBLE, extended-spectrum ß-lactamase-producing Enterobacteriaceae; VAP, ventilator-associated pneumonia

The following factors were significantly associated with higher rates of ESBLE VAP by univariate analysis: female gender (p = 0.04), medical admission (p = 0.03), ARDS (p = 0.01), shock (p = 0.02), infection at ICU admission (p = 0.04), and digestive tract colonization related to ESBLE (p < 0.001). Male gender, and acute exacerbation of COPD at ICU admission were significantly associated with lower rate of ESBLE VAP by univariate analysis (Tables 1, and 3).

Multivariate analysis identified prior ESBLE colonization of the digestive tract as the only independent risk factor for ESBLE VAP (OR [95% CI] = 23 [10–55], p < 0.001) (Table 5). Sensitivity and specificity of prior digestive ESBLE colonization as a predictor of ESBLE VAP were 79% (95% CI = 64.0–84.0) and 88% (95% CI = 84.2–91.2). Whilst the positive predictive value of ESBLE digestive colonization was low (43.6%), its negative predictive value was excellent (97.3%) in predicting ESBLE VAP.

**Table 5:** Risk factor for ventilator-associated pneumonia related to ESBLE by multivariate analysis.

Female gender	0.80	0.90 [0.39–2.07]
**Medical admission**	0.33	0.61 [0.23–1.66]
**Acute exacerbation of COPD**	0.06	0.13 [0.02–1.12]
**Acute respiratory distress syndrome**	0.31	1.76 [0.59–5.29]
**Shock**	0.40	1.43 [0.62–3.33]
**Infection at admission**	0.15	2.81 [0.70–11.32]
**Prior antibiotic exposure**	0.84	1.10 [0.44–2.71]
**Broad-spectrum antibiotics exposure**	0.93	1.05 [0.35–3.13]
**Prior digestive colonization**	< 0.001	23.32 [9.89–54.97]

COPD, chronic obstructive pulmonary disease

### Outcomes

No significant difference was found in duration of mechanical ventilation (median [IQR], 28 [18,42] vs 23 [15,42] d, p = 0.4), length of ICU stay (31 [19,53] vs 29 [18,46] d, p = 0.6), and mortality rates (55.8% vs 50%, p = 0.48) between the two groups (Table 6). No significant difference was found in time from VAP diagnosis to death between patients with ESBL VAP, and those with VAP related to other bacteria (10 [4,27] days (median [IQR]) versus 12 [5,24] respectively, p = 0.48).

**Table 6:** Patient outcomes.

Variable	ESBLE VAP n = 43	No ESBLE VAP n = 367	*p*
**Duration of mechanical ventilation, days**	28 [18,42]	23 [15,42]	0.4
**ICU length of stay, days**	31 [19,53]	29 [18,46]	0.6
**ICU mortality**	24 (56)	184 (50)	0.48

Results by univariate analysis. Data are presented as number (%) or median (interquartile range).

ESBLE, extended-spectrum ß-lactamase-producing Enterobacteriaceae; VAP, ventilator-associated pneumonia; ICU, intensive care unit.

## Discussion

Our results suggest that prior digestive colonization with ESBLE is independently associated with occurrence of VAP related to ESBLE. The negative predictive value of prior digestive colonization to detect VAP due to ESBLE was excellent, although the positive predictive value was low. No significant difference was found in duration of mechanical ventilation, ICU length of stay or mortality between patients with ESBLE VAP, as compared with those with VAP due to other bacteria.

Several previous studies identified risk factors for MDR bacteria and ESBLE colonization [13,14,18–24]. However, few specific data are available about VAP related to ESBLE. Bruyère et al. performed a single center retrospective study, over the same time period, to assess the interest of screening for ESBLE rectal carriage to predict their involvement in VAP [25]. Their study included 587 patients with suspected VAP. VAP was caused by ESBLE in 20 (3.4%) patients, and among them 17 were previously identified as ESBLE carriers. Sensitivity and specificity of prior ESBLE colonization as a predictor of ESBLE VAP were 85.0% and 95.7%, respectively. The negative predictive value was 99.4%. Our results confirm their findings in a cohort with higher number (n = 43) of patients with VAP related to ESBL. Our results suggest that patients without ESBLE colonization should probably not receive carbapenems as part of their initial empirical treatment to cover ESBLE. Such a strategy of restricting the use of carbapenems would be helpful to prevent subsequent resistance.

Most cases of VAP result from aspiration of oropharyngeal, or gastric secretions around the tracheal-tube cuff into the normally sterile lower respiratory tract [26,27]. Digestive tract represents a potential reservoir of nosocomial Gram-negative bacilli, which may become a source of colonization of the respiratory tract during mechanical ventilation. Gram-negative bacilli, such as *Pseudomonas aeruginosa* and *Klebsiella pneumonia* are the most common microorganisms in patients with VAP [2,28]. Recent studies reported a substantial increase in the incidence of ESBLE in community and in ICU patients, in several countries, including France [20–22,29].

In our population, 297 patients (72.4%) acquired MDR bacteria during their ICU stay and among them, digestive tract colonization with ESBLE was found in 125 patients (30.5%). This result could be explained by the high percentage of patients with late-onset VAP (80%), the long duration of ICU stay and mechanical ventilation in study patients, and the high proportion of patients who received broad-spectrum antibiotic treatment.

Previous use of broad-spectrum antibiotics, including beta-lactam/beta-lactamase and carbapenems was identified in many studies as a risk factor for ESBLE colonization [14,24,30–32]. In addition, previous studies reported that digestive tract ESBLE colonization was associated with an increased mortality [14]. In our study, no significant difference was found in prior exposure to broad spectrum antibiotic treatment, or to carbapenems between ESBLE VAP, as compared to VAP related to other bacteria. Further, ESBLE VAP was not associated with negative impact on outcome. These results could be explained by the relatively small number of patients with ESBLE VAP.

Our results also suggest that a systematic screening strategy for ESBLE digestive colonization during the ICU stay is probably helpful to guide empirical antibiotic treatment in VAP patients. Nine patients developed an ESBLE VAP without prior digestive tract colonization. In three of them, ESBLE digestive colonization was detected thereafter. Screening by rectal swab twice a week could have improved sensitivity [24]. Nevertheless, increasing the screening frequency could overestimate the risk of ESBLE VAP and the potential use of carbapenems [25].

Our study has some limitations. First, it was a retrospective study, although VAP were prospectively identified. It was also performed in a single center. Therefore, our results could not be generalized. Second, only the first episodes of VAP were taken into account in our study. Some patients were exposed to prolonged duration of mechanical ventilation and developed several episodes of VAP requiring antimicrobial treatment. Therefore, the incidence of ESBLE VAP might have been underestimated, as late-onset subsequent VAP episodes were not studied. Third, because of the retrospective design, appropriateness of empirical antibiotic treatment could not be evaluated. Fourth, we did not collect information on PaO_2_/FiO_2_ ratio in study patients. Fifth, regarding our sample size (n = 410) and number of patients with ESBLE VAP (n = 43), we caution that we cannot not exclude that several differences were overlooked due to the lack of adequate statistical power, and that we cannot exclude the issue of overfitting in multivariate analysis. Finally, one could argue that a control group including only patients with VAP related to Enterobacteriaceae-other than ESBL could be more relevant than VAP related to bacteria-other than ESBL. However, at the time of VAP suspicion, no information is available on the responsible microorganism. In addition, we repeated all analyses using a control group with VAP related to Enterobacteriaceae-other than ESBL, and found similar results (data not shown).

## Conclusion

Our results suggest a significant relationship between digestive tract colonization related to ESBLE and the occurrence of ESBLE VAP. The excellent negative predictive value suggests that patients without ESBLE colonization should not receive carbapenems as part of their initial empirical treatment to cover ESBLE. However, further prospective studies are required to confirm our results.
